# Supplementary material for: Strong modulation of second-harmonic generation with very large contrast in semiconducting CdS via high-field domain
Source: Nat Commun. 2018 Jan 15;9:186. doi: 10.1038/s41467-017-02548-3 (PMC5768866; doi:10.1038/s41467-017-02548-3)
Supplement: Supplementary file 1 — Supplementary Materials [file 41467_2017_2548_MOESM1_ESM.pdf]

Supplementary Note 1

Analysis of the field induced nonlinear coefficients

CdS belongs to the point group 6mm and only exhibits non-zero second-order nonlinear coefficients or tensor elements ($\chi^{(2)}$-elements), $\chi^{(2)}_{xzx}$ (d_{15}), $\chi^{(2)}_{xxy}$ (d_{24}), $\chi^{(2)}_{zzx}$ (d_{31}), $\chi^{(2)}_{zyy}$ (d_{32}), and $\chi^{(2)}_{zzz}$ (d_{33}). Without any loss of generality, we assume the excitation laser propagates along the y-axis and only consider field components in the xz-plane. Moreover, the third-order nonlinear coefficients or tensor elements ($\chi^{(3)}$-elements) are, $\chi^{(3)}_{xxxx}$, $\chi^{(3)}_{xzzx}$, $\chi^{(3)}_{zxzx}$, $\chi^{(3)}_{xzxz}$, $\chi^{(3)}_{zxxz}$ and $\chi^{(3)}_{zzzz}$, which will contribute to SHG via the interplay with an external field. In CdS, SHG is expressed by the following formula[1],

$$\begin{pmatrix} E_{2\omega,x} \\ E_{2\omega,y} \\ E_{2\omega,z} \end{pmatrix} = \begin{pmatrix} 0 & 0 & 0 & 0 & d_{15} & 0 \\ 0 & 0 & 0 & d_{24} & 0 & 0 \\ d_{31} & d_{32} & d_{33} & 0 & 0 & 0 \end{pmatrix} \begin{pmatrix} E^2_{\omega,x} \\ E^2_{\omega,y} \\ E^2_{\omega,z} \\ 2E_{\omega,y}E_{\omega,z} \\ 2E_{\omega,z}E_{\omega,x} \\ 2E_{\omega,x}E_{\omega,y} \end{pmatrix}, \quad (1)$$

Here $E_{\omega,i}$ (or $E_{2\omega,i}$) depicts the i-component (i=x, y, z) of electric field of FW (or SHG). In terms of electric field induced second-harmonic generation (EFISH), the second-order nonlinear coefficient is rewritten as,

$$\chi^{(2)}_{ijk} = \chi^{(2)}_{ijk,0} + \chi^{(3)}_{ijkl} F_l, \quad (2)$$

where, $\chi^{(2)}_{ijk,0}$ stands for the nonlinear coefficient with no applied field and is zero for structures with mirror symmetry, and F_l represents the applied field. Therefore, if the applied field is along the x-axis of CdS (F_x), the new nonlinear coefficients are induced,

$$\chi^{(2)}_{xxx} = \chi^{(3)}_{xxxx} F_x,\ \chi^{(2)}_{xzz} = \chi^{(3)}_{xzzx} F_x,\ \text{and}\ \chi^{(2)}_{zzx} = \chi^{(3)}_{zzxx} F_x = \chi^{(2)}_{zxz} = \chi^{(3)}_{zxzx} F_x, \qquad (3)$$

A new nonlinear coefficient means it is intrinsically zero and will be produced by applying an external stimulus. In this work, we focus on $d_{11} = \chi^{(2)}_{xxx} = \chi^{(3)}_{xxxx} F_x$.

To determine the cystography of the CdS nanobelt, we performed SHG polarimetry[2]. As shown in Supplementary Fig. 1, the a-axis (or x-axis) is along the long axis of the nanobelt.

To estimate the error of measured d_{11}, we looked at the power of the fundamental light, P_ω, which was measured by the laser power meter (LabMax-TO, Coherent). Its fluctuation is estimated to be $\Delta\sim\pm0.6\%$, causing the actual pump power to be $P_\omega*(1\pm\Delta)$. When using d_{33} to estimate d_{11}, we fixed the average pump power to measure d_{33}-SHG and d_{11}-SHG and extracted d_{11} as follows,

$$d_{11} = d_{33}\sqrt{\frac{P_{2\omega}(d_{11})}{P_{2\omega}(d_{33})}}\times\frac{P_\omega(d_{33})}{P_\omega(d_{11})} = 151\times\frac{1\pm\Delta}{1\pm\Delta} \approx 151\times(1\pm2\Delta)$$

Therefore the error or uncertainty of d_{11} is $2\times0.6\%\times151$ pmV^{-1} = ±1.8 pmV^{-1}, i.e., $d_{11}=151\pm1.8$ pmV^{-1}.

Note that $d_{11}=151$ pmV^{-1} and $\chi^{(3)}_{xxx}=1.1\times10^{-17}$ m^{2}V^{-2}, we can estimate the electric field strength using $F_c = d_{11}/\chi^{(3)}_{xxx}$. It is ~130 KVcm^{-1} which is below the damage threshold of CdS (~150 KVcm^{-1}).

Supplementary Note 2

Analysis of Schottky contacts in our CdS nanobelt device

For simplicity, we only consider completely ionized donors contributing to the space charge in a classic Schottky barrier, and obtain the potential (V) by solving the Poisson equation[3, 4],

$$-\frac{d^2V}{dx^2}=\frac{dF}{dx}=\begin{cases}\dfrac{\rho}{\varepsilon_r\varepsilon_0}=h,(0\le x\le x_d)\\ 0 \quad\quad ,(x>x_d)\end{cases} \tag{4}$$

where x_d is the width of the Schottky barrier, $\rho=qN_D$ is the space charge, N_D is the donor density, ε_0 is the dielectric in vacuum, and ε_r is the dielectric constant of CdS. By assuming that the internal field is zero in the bulk semiconductor, $F(x_d)=-dV(x_d)/dx=0$, we obtain the internal electric field and current,

$$F(x)=h(x-x_d), \tag{5}$$

$$J_{DS}=J_{s0}[\exp(\frac{qV}{k_0T})-1], \tag{6}$$

where

$$x_d=\{-\frac{2\varepsilon_r\varepsilon_0[V_{s0}+V]}{qN_D}\}^{1/2}=\{\frac{2\varepsilon_r\varepsilon_0[V_{in}-V]}{qN_D}\}^{1/2} \tag{7}$$

$$J_{s0}\propto\{2h(V_{in}-V)\}^{1/2}\exp(-\frac{qV_{in}}{k_0T}), \tag{8}$$

V_{s0} is the surface potential and is negative for n-type CdS, the built-in potential V_{in}= -V_{s0}. If the Schottky barrier is positively biased on the metal (V>0), the forward current (J_{DS}) grows

exponentially with respect to voltage whereas the barrier width (x_d) and field at the interface (x=0) decrease. If V<0, the reverse current approaches $-J_{s0}$ with respect to voltage. In this case, the Schottky barrier increases in its width and internal field at each position.

To interpret the current response to the applied voltage or I-V curve (Fig. 2B), we assume the Schottky contacts, i.e., drain (D) and source (S), are asymmetric ($V_{DD} >> V_{SS}$). Here the built-in potential V_{DD} (>0) and V_{SS} (>0) corresponds to the D and S contacts respectively (Fig. 1C inset and Supplementary Fig. 2A). Typically, V_{DD} and V_{SS} <<k_0T/q ~23 meV at 300 K. When a positive voltage (V_{DS}>0) is applied on contact D (Supplementary Fig. 2B), the Schottky contact D is positively biased whereas the contact S is reversely biased. In this case, the forward current (J_{DS}, from contact D to S) is first determined by the contact D since $V_{DD} >> V_{SS}$ and grows exponentially as described in Supplementary Equation 6. As the positive voltage increases, the barrier height of the contact D decreases ($V_{DD} - V_D \to 0$) whereas the barrier height of the contact S increases ($V_{SS} + V_S >> V_{DD} - V_D$) and becomes dominant in J_{DS} (i.e. J_{s0} in Supplementary Equation 8 and Supplementary Fig. 2B). Thus, the curvature of I-V curve is changed from convex ($d^2J_{DS}/dV^2 > 0$, forward current relative to the contact D, as shown in Supplementary Equation 6) to concave ($d^2J_{DS}/dV^2 < 0$, reverse current relative to the contact S, i.e., J_{s0} (V_{SS}+V_S) in Supplementary Equation 8) with the applied voltage, as observed in Fig. 2B. Here $V_{DS} = V_D + V_S$, V_D is the voltage applied on the contact D whereas V_S is applied on the contact S. When the internal electric field in the contact S approaches a critical field, the field induced ionization of acceptor traps becomes significant and then initiates the high-field domain at the contact S (discuss latter), whereby the current saturates, as shown in Fig. 2B.

If a negative voltage (V_{DS}<0) is applied upon the contact D (Supplementary Fig. 2C), the contact D is reversely biased whereas the contact S is now forward biased. Since $V_{DD} >> V_{SS}$, the contact D is more significant to control the reverse current (J_{DS}=J_{s0} (V_{DD}+V_D) <0, reverse current relative to the contact D, direction from S to D) (Fig. 4B and Supplementary Fig. 2C). In this case, an increase in applied voltage will increase the internal field and width near the contact D. When the internal field approaches a critical field, the high-field domain can be induced near the contact D (Fig. 4A). The barrier height near the contact S decreases and has little impact on the I-V curve.

Supplementary Note 3

Schottky high-field domain model based on field induced ionization of acceptor traps

The high-field domain has been observed in CdS owing to field induced ionization of traps[3, 5-7]. As known, CdS is *n*-type due to a large number of sulfur vacancies or electron traps or donors and the ionized donor traps are positively charged with a density of N_D. In our case, there are also a large number of deep-level neutral accepter traps (cadmium vacancies) in CdS which can accept electrons and become negatively charged [8]. If a large electric field is applied, electrons can be excited from the valence band into accepter traps [4]. Therefore acceptor traps will be ionized and negatively charged with the density N_A and contribute to the space charge (N_D-N_A). The ionized holes will be free in the valence band to recombine with electrons in the conduction band and can also participate in conduction via delocalized band states. This process is called field induced ionization of acceptor traps. If the density (N_A) of the ionized acceptor traps induced by a field (F_c) is increased up to the density (N_D) of the ionized donor traps, i.e.,

$$N_D - N_A = 0. \tag{9}$$

the net space charge is $\rho=q(N_D-N_A) = 0$ and the internal field remains constant at F_c (Supplementary Equation 4) and current saturates. F_c is the critical field under which Supplementary Equation 9 is satisfied. The region of field induced space charge neutrality is the so called high-field domain with a constant field of F_c [5, 7]. Since the high-field domain is initialized near the reversely-biased Schottky barrier, we regard this model as Schottky high-field domain model. Please see Supplementary Fig. 3A for distributions in the electric field and net space charge.

To explain our experiments using Schottky high-field domain model, we assume V_c is the critical voltage at which the internal field approaches the critical field F_c and $B(V_b)$ depicts the Schottky barrier at the applied voltage V_b. Given a positive voltage ($V=V_{DS}>0$) applied upon the contact D (the drain contact), the high-field domain is formed in the following steps,

(1) When $V<V_c$, an increase of the applied voltage (V) will increase the internal field (F) and width (x_d) of the Schottky barrier S (Supplementary Equations 5 and 7). In this case, the Schottky barrier is B ($V_b=V<V_c$).

(2) When $V=V_c$, $F=F_c$ at the metal-semiconductor interface of the contact S. Supplementary Equation 9 is satisfied and the high-field domain is initialized at the interface. Its width is small ($x_c \approx 0$). The barrier is retained at $F\approx F_c$ at $x=x_c$ and $F=0$ at $x = x_d+x_c$. Since the applied voltage ($V=V_c$) is also applied upon the formation of the high-field domain, the Schottky barrier is still $B(V_b=V_c-F_c x_c<V_c)$.

(3) When $V>V_c$, an increase of applied voltage will mostly be applied upon the Schottky barrier and drive the high-field domain to expand into a new region with the constant

field F_c following steps (1) and (2). This process extends the high-field domain by $x_c \approx (V - V_c)/F_c$ and rebuilds the barrier at $x=x_c$.

Similarly, at a negative voltage ($V=V_{DS}<0$), the HFD is initiated at the contact D which is reversely biased and expands toward to the contact S as applied voltage increases.

Once the high-field domain is initiated, an increase in $V=V_{DS}$ only extends the high-field domain by $(V-V_c)/F_c$, and the electric field at any arbitrary position (x) is given by (Fig. 2D),

$$F(x)=\begin{cases}-F_c, & x<x_c \\ -F_c+h(x-x_c), & x_c \le x \le x_d+x_c, \\ 0, & x_d+x_c<x<L\end{cases} \tag{10}$$

where $x_c=(V-V_c)/F_c$, $x_d=F_c/h$ and L is the sample length, assuming that $x_d+x_c<L$. The region of $x<x_c$ is the high-field domain while the region of $x_c \le x < x_d+x_c$ is the barrier or domain boundary. It is clearly shown that if $x_0<(V-V_c)/F_c$, the point at $x=x_0$ has already been within the high-field domain and the field should be $-F_c$.

Supplementary Note 4

High-field domain induced SHG

In our experiment, the fundamental wave (FW) is a Gaussian beam,

$$g(x,x_0,w)=\frac{1}{\sqrt{2\pi}\sigma}\exp[-(\frac{x-x_0}{\sqrt{2}\sigma})^2], \tag{11}$$

where x_0 is the centre of the laser spot and $\sigma = w/2$ with the beam waist of $2w$ ~3 μm in our case.

Therefore, the SHG field excited from the excitation region (centred at x_0) is,

$$
\begin{aligned}
E_{2\omega}(x_c, x_0) &= \chi^{(3)}_{xxxx} I_\omega [\int_0^L F(x - x_c) g(x, x_0, w) dx] \\
&= \chi^{(3)}_{xxxx} I_\omega F_c [\int_0^L f(x - x_c) g(x, x_0, w) dx] \\
&= \chi^{(3)}_{xxxx} I_\omega F_c [\int_0^{x_d + x_c} -g(x, x_0, w) dx \\
&\qquad + \frac{h}{F_c} \int_{x_c}^{x_d + x_c} (x - x_0) g(x, x_0, w) dx \\
&\qquad - \frac{h}{F_c} (x_c - x_0) \int_{x_c}^{x_d + x_c} g(x, x_0, w) dx] \\
&= \chi^{(3)}_{xxxx} I_\omega F_c [A + B + C],
\end{aligned}
\tag{12}
$$

where I_ω is the intensity of FW, the profile of the internal field $f(x) = F(x)/F_c$ and

$$A = \int_0^{x_d + x_c} -g(x, x_0, w) dx = -\frac{1}{2} [erf(\frac{x_d + x_c - x_0}{\sqrt{2}\sigma}) - erf(-\frac{x_0}{\sqrt{2}\sigma})], \tag{13}$$

$$B = \frac{h}{F_c} \int_{x_c}^{x_d + x_c} (x - x_0) g(x, x_0, w) dx = -\sqrt{\frac{1}{2\pi}} \frac{h\sigma}{F_c} \{\exp[-(\frac{x_d + x_c - x_0}{\sqrt{2}\sigma})^2] - \exp[-(\frac{x_c - x_0}{\sqrt{2}\sigma})^2]\}, \tag{14}$$

$$C = -\frac{h}{F_c} (x_c - x_0) \int_{x_c}^{x_d + x_c} g(x, x_0, w) dx = -\frac{h(x_c - x_0)}{2F_c} [erf(\frac{x_d + x_c - x_0}{\sqrt{2}\sigma}) - erf(\frac{x_c - x_0}{\sqrt{2}\sigma})]. \tag{15}$$

Where $erf(x)$ is the error-function and defined as,

$$erf(x) = \frac{2}{\sqrt{\pi}} \int_0^x \exp[-t^2] dt, \tag{16}$$

If we consider a simple case without the domain boundary (or Schottky barrier), the field profile is a step function,

$$F(x)=\begin{cases}-F_c, & x<x_c \\ 0, & x>x_c\end{cases}, \tag{17}$$

Then the SHG field is,

$$\begin{aligned}E_{2\omega}(x_c,\ x_0) &= \chi^{(3)}_{xxxx}I_\omega[\int_0^L F(x-x_0)g(x,x_0,w)dx] \\ &= \chi^{(3)}_{xxxx}I_\omega F_c\int_0^{x_c} g(x,x_0,w)dx=\frac{1}{2}\chi^{(3)}_{xxxx}I_\omega F_c[erf(\frac{x_c-x_0}{\sqrt{2}\sigma})+erf(\frac{x_0}{\sqrt{2}\sigma})].\end{aligned} \tag{18}$$

The SHG intensity is calculated from Supplementary Equation 12,

$$I_{2\omega}(x_c,x_0)=E^2_{2\omega}(x_c,x_0)=[\chi^{(3)}_{xxxx}F_cI_\omega G(x_c,x_0,w)]^2, \tag{19}$$

where $G(x_c,x_0,w)=A+B+C$.

In Supplementary Equations. 13-16, if $x_c=x_0$,

$A=-\frac{1}{2}[erf(\frac{x_d}{\sqrt{2}\sigma})+erf(\frac{x_0}{\sqrt{2}\sigma})], B=-\sqrt{\frac{1}{2\pi}}\frac{h\sigma}{F_c}\{\exp[-(\frac{x_d}{\sqrt{2}\sigma})^2]-1]\}$ and $C=0$, and

$G(x_c,x_0,F_c)=-\frac{1}{2}\{erf(\frac{x_d}{\sqrt{2}\sigma})+erf(\frac{x_0}{\sqrt{2}\sigma})+\sqrt{\frac{2}{\pi}}\frac{h\sigma}{F_c}\exp[-(\frac{x_d}{\sqrt{2}\sigma})^2]-\sqrt{\frac{2}{\pi}}\frac{h\sigma}{F_c}\}$.

if $x_c\to+\infty$, $A=-\frac{1}{2}[1+erf(\frac{x_0}{\sqrt{2}\sigma})], B=0$ and $C=0$, and

$G(x_c, x_0, F_c) = \frac{1}{2}[1 + erf(\frac{x_0}{\sqrt{2}\sigma})]$ is independent of F_c and the SHG signal saturates.

Supplementary Note 5

Effect of near infrared (IR) radiation on high-field domain

As discussed in Supplementary Note 4, deep-level acceptor traps (E_A above the mid-gap) are ionized or filled when the high-field domain is initialized. If IR light (e.g. at λ_ω=1018 nm) is illuminated upon the high-field domain, electrons in the acceptor traps can be excited into the conduction band and then take part in conduction. Due to the one-photon excitation process (E_C-E_A<hc/λ_ω), the change of the current is linearly proportional to the IR intensity (I_ω) since $\Delta J \propto \Delta n \propto I_\omega$, as observed (Supplementary Fig. 4B). Here Δn is the density of electrons that are excited from the deep-level acceptor traps via the IR excitation. In order to understand the effect of IR excitation on the high-field domain, we assume an electron from the valence band is excited into the acceptor trap via field emission with the rate $\gamma(F_c)$ [4], and an electron in the acceptor trap is pumped out via the IR excitation with the rate γ_{IR}. Here γ_{IR} is linearly proportional to I_ω. In steady-state, electrons that are pumped into the acceptor traps should equal the electrons that are pumped out, therefore the change of the field emission rate $\Delta\gamma(F_c)$,

$$\Delta\gamma(F_c) = \gamma_{IR}. \tag{20}$$

Note that $\Delta\gamma(F_c)=\gamma(F_c)-\gamma(F_{c0})=\frac{d\gamma(F_{c0})}{dF}\Delta F_c+\frac{1}{2}\frac{d^2\gamma(F_{c0})}{dF^2}\Delta F_c^2+\cdots$ and $\gamma_{IR}\propto I_\omega$, we assume the change of the field strength $\Delta F_c=F_c-F_{c0}$ is very small and ignore the high-order terms and obtain,

$$\Delta F_c=\alpha I_\omega, \tag{21}$$

where α is the coefficient which is independent of the IR intensity and relates to the electric field strength (F_{c0}) of the high-field domain without IR excitation. Hence, the normalized conversion efficiency of SHG is,

$$\eta_{2\omega}\propto F_c^2=(F_{c0}+\Delta F_c)^2=(F_{c0}+\alpha I_\omega)^2\approx F_{c0}^2+2F_{c0}\alpha I_\omega, \tag{22}$$

and clearly shows linear response to the IR intensity, as observed in our experiments (Fig. 4C and Supplementary Fig. 4D).

Field-induced SHG can be calculated using Equation 1 and Supplementary Equation 19. The saturation voltage (V_s) and threshold voltage (V_t) are extracted from the voltage dependence of SHG and the critical voltage (V_c) is referred to the I-V curve. To clarify our calculations, we use Fig. 4A as an example. The excitation region remained unchanged at x_0 when the excitation intensity is varied. As both saturation voltage and critical field (F_c) are linearly varied with the excitation intensity (Supplementary Fig. 4D and 4E), it is reasonably assumed that the critical field is linearly correlated with the critical voltage (V_c) given $V_s\approx V_c+F_c x_0$. In our calculations, we chose donor concentration N_D=5×10^{21} m^{-3}, interface electron density n_c = 0.1N_D, electron mobility μ_n=350 cm^{2}Vs^{-1}, and temperature T=300 K, ε_r=8.9 for CdS [7] and fitted the voltage dependence of SHG to extract the critical field. As shown in Fig. 2A and Fig. 4A, our calculated

SHG is fit to measured voltage dependences of SHG very well. To calculate SHG in Fig. 4D, we first extracted the relationship between the critical field and excitation intensity from Fig. 4A. Then we substituted the critical field into Equation 1 and Supplementary Equation 19 to perform calculations given the excitation intensity and the -30 V bias. The fitting is shown to be good in Fig. 4D.

Supplementary Note 6

Characteristics of CdS SHG transistor

Typically, one characterizes a transistor using ON/OFF ratio, mobility, subthreshold slope and/or modulation strength. For the CdS SHG transistor device, we do it in a similar way. Analogous to the characteristic of the drain current of metal-oxide-semiconductor field effect transistor (MOSFET) at the subthreshold region [4], we define the subthreshold slope (k_s) for the SHG transistor, which indicates the transition rate (fast or slow) between OFF (low signal) and ON (high signal) states as a function of bias.

$$k_s = \frac{d\log(I_{2\omega})}{dV} = \frac{1}{I_{2\omega}}\frac{dI_{2\omega}}{dV}. \tag{23}$$

Besides, the ON/OFF ratio is defined as the ratio between SHG intensities in the ON and OFF states. The ON state corresponds to the saturation SHG (SHG_{max}), for example the SHG intensity at ~57 V in Fig. 1C. The OFF state is the SHG intensity (SHG_{min}) at ~22 V where SHG starts to appear and can be reliably recorded above the noise floor. The ON/OFF ratio is SHG_{max}/SHG_{min}. Please note that the OFF value will be more accurate and smaller if using a more sensitive detector. If the ON/OFF ratio is very large (approaching infinity), then

modulation contrast is a more meaningful metric of the device. It is defined as (SHG_{max}-SHG_{min})/(SHG_{max}+SHG_{min}). If SHG_{max}>> SHG_{min}, the modulation contrast approaches 1, which is the case of perfect contrast. If SHG_{max}~ SHG_{min}, the modulation contrast approaches 0 and no modulation is achieved.

By analyzing the fitted parameters in Fig. 2A, we can characterize this CdS SHG transistor under 1018 nm excitation and obtained a very large value of ON/OFF ratio of 10^4, steep subthreshold slope ($k_s = d\log I_{2\omega}/dV$) of ~200%$V^{-1}$ and near perfect contrast of 1. More details can be found in Supplementary Fig. 6. The subthreshold slope corresponds to the modulation strength of SHG in the field-related devices and is ~20 times larger than other reports (<10%V^{-1})[9-11]. The steep subthreshold slope or modulation strength indicates fast transition between the OFF and ON states, demonstrating the enhanced performance of our SHG transistor.

Other studies have also been reported regarding electric-field induced SHG[12] and estimation of newly generated second-order nonlinear coefficients [13].

Supplementary Note 7

Estimation of the response time of device

As described in the main manuscript, the high-field domain is due to field-enhanced ionization of traps. This is the process of driving electrons from the valence band to acceptor traps (i.e. deep-level Cd vacancies) via electric field. Note that the cross-section of capturing holes is ~10^{-17} cm^2 for these acceptor holes [8]. In order to achieve high-field domain, the large cross-section for capturing electrons (>10^{-17} – 10^{-18} cm^2) is required to ionize these acceptor traps and can be

achieved by electric field. Considering the density of acceptor traps N_A ~10^{16} -10^{17} cm^{-3} [8], thermal velocity of electron <v> ~10^7 cm s^{-1} and the cross-section of capturing electrons S_c >10^{-17} cm^2, the response time of acceptor traps can be estimated as [8],

$$\tau = \frac{1}{S_c N_A < v >} < 1\mu s$$

However, this estimation can become significantly less, leading to a much faster response from the device due to strong applied fields which strongly modulates carrier trapping/de-trapping rates. Also, improved device geometries, smaller cross-section, operating close to the threshold via applying a d.c voltage below the threshold and a modulating voltage to cross the threshold, better spatial overlap, smaller spot size can significantly improve the switching times.

Supplementary Note 8

Heating effect on d_{11}-SHG signals

When the current is high (e.g. 4 kA cm^{-2} in Fig. 2B), the heating effect could be introduced and influence the intensity of d_{11}-SHG signals. It will cause the shift of exciton resonances and may lead to changes in third-order nonlinear coefficients. It may also affect the formation of high-field domain by altering the carrier dynamics, such as the decay rate of carriers in acceptor traps. To evaluate the impact of heating, we performed the following experiments. We tested the device at 77 K and observed d11-SHG signals which behave similar to 300K (e.g. saturation, Supplementary Fig. 7A). Heating will not change the origin of d_{11}-SHG. Then we heated the device with a heater (300K to 340K) and did not observe any d11-SHG signals (see below) without a bias voltage, showing the heating cannot produce d_{11}-SHG signals. Please see more

details in Supplementary Fig. 7B. Moreover, pump power dependence of d_{11}-SHG shows that with an increase in pump power will increase the saturation current and d_{11}-SHG when the excitation light is focused onto the high-field domain (see Fig. 4C). This shows the heating effect due to high current would have positive impact on d_{11}-SHG. Gate voltage dependence of d_{11}-SHG shows that increase in gate voltage will increase current and decrease d_{11}-SHG (see Fig. 4E). This shows the heating effect due to high current would have a negative impact on d_{11}-SHG. Therefore, combining pump power and gate voltage dependence, it can be seen that heating does not significantly alter d_{11}-SHG signals.

Supplementary Note 9

Discussion on Phase Mismatch in CdS nanobelts

Typically, the SHG field can be written as [1],

$$E_{2\omega} = A\frac{\sin{(\Delta kl/2)}}{\Delta kl/2} = Asinc\left(\frac{\Delta kl}{2}\right) = Asinc\left(\frac{\pi}{2}\frac{l}{l_c}\right), \tag{24}$$

where A is related to the electric field of the fundamental wave, nonlinear coefficient and sample length (l), $\Delta k = k_{2\omega} - 2k_{\omega} = \pi/l_c$ is the phase mismatch between the second-harmonic (SH) wave and fundamental wave, l_c is the coherence length, and $k_{2\omega}$ (or k_{ω}) is the wave-vector of the second-harmonic wave (or fundamental wave), $sinc(\Delta kl/2)$ is the factor of the phase mismatch.

When phase mismatch (i.e. $\Delta k \neq 0$) occurs between the second-harmonic (SH) wave and fundamental wave, the SH wave is converted from the fundamental wave in the first coherence length and converted back to the fundamental wave in the second coherence length. This is one

cycle and will be repeated if the sample is very long. If the phase matching is satisfied (i.e. $\Delta k = 0$), the SH wave will always be converted from the fundamental light and grow continuously. This is why the phase matching is very important to achieve high conversion efficiency in bulk materials. In our work, the phase mismatch effect was not considered in the theoretical model because it does not induce SHG. It only contributes to the signal intensity after SHG was induced. This effect was automatically considered in the SHG signal (e.g. d_{11}-SHG) measured in our experiments, but it is not significant and has no impact on our reported results. First of all, the sample thickness is only few hundred nanometers (l ~300 nm) and well within one coherence length (l_c ~800-900 nm). Given Supplementary Equation 24, the SHG field generated in our case is ~5.5% lower than the assumed case of phase matching. Hence the phase mismatch effect is not significant. Secondly, the effective nonlinear coefficient, d_{11}, is obtained by comparing d_{11}-SHG with intrinsic d_{33}-SHG. Therefore, signal intensity reduction due to phase mismatch is cancelled out (i.e. d_{11}-SHG/d_{33}-SHG) and has little impact on d_{11}. Thirdly, the voltage dependence of SHG is normalized and fitted to the model. The factor of phase mismatch will not have impact on the behaviour or trend. Therefore, it is common to ignore the phase mismatch issue in nanostructures[10].

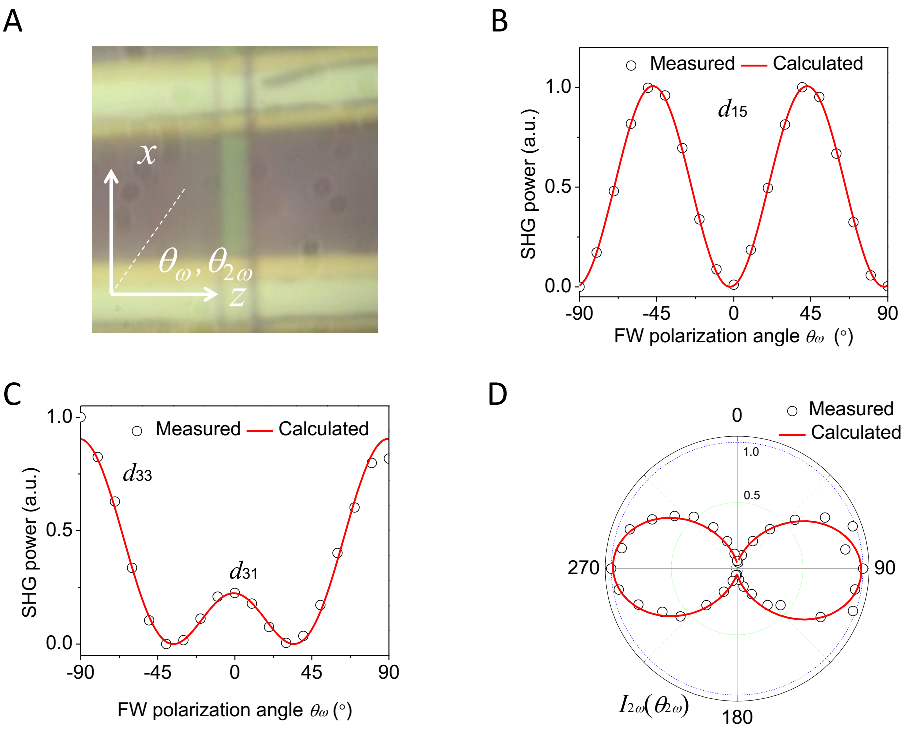

Supplementary Figure 1. **Orientation dependent second-harmonic generation (SHG) signal from the CdS nanobelt at zero applied bias**. **A**, Optical image of the CdS nanobelt. θ_ω (or $\theta_{2\omega}$) stands for the polarization angle of fundamental wave (FW) (or SHG) with respect to the *x*-axis. **B**, *x*-polarized SHG (d_{15}) as a function of the polarization angle of FW, relating to second order nonlinear coefficient d_{15}. **C**, *z*-polarized SHG in response to the polarization angle of FW, involving d_{31} and d_{33}. **D**, Polarmetric SHG when FW is polarized along the *x*-axis.

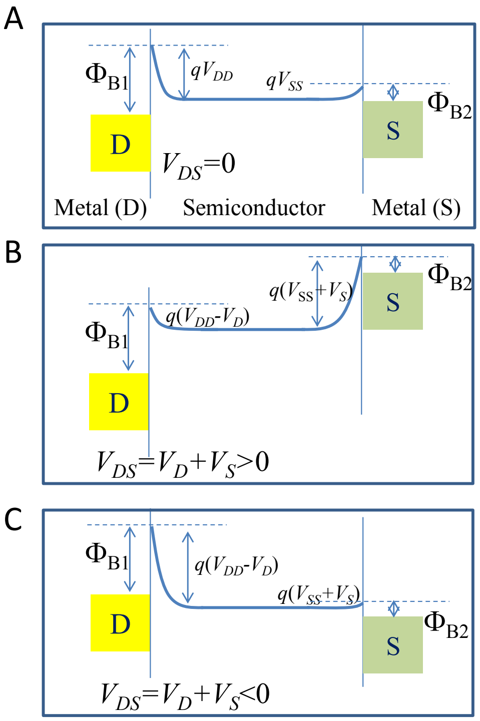

Supplementary Figure 2. Band diagram of the metal-semiconductor-metal structure in a CdS device. V_{DD} (or V_{SS}) is the barrier height near the D (or S) contact. Owing to different metals (Au and Ti) for different contacts (Fig. 1A), the contacts (D and S) have different barrier heights (V_{DD}>>V_{SS}). Φ_{B1} is the potential difference between Au and CdS near the D contact while Φ_{B2} is the potential difference between Ti and CdS near the S contact. **A** bias voltage (V_{DS}) is applied on the contact D. V_D (or V_S) is the bias voltage applied on the D (or S) contact. A, Band structure when V_{DS}=0. **B**, Band structure when V_{DS}>0. The contact D is forward biased with V_D (>0) and contact S is reversely biased with V_S (>0). **C**, Band structure when V_{DS}<0. The contact D is reverse biased with V_D (<0) and contact S is forward biased with V_S (<0).

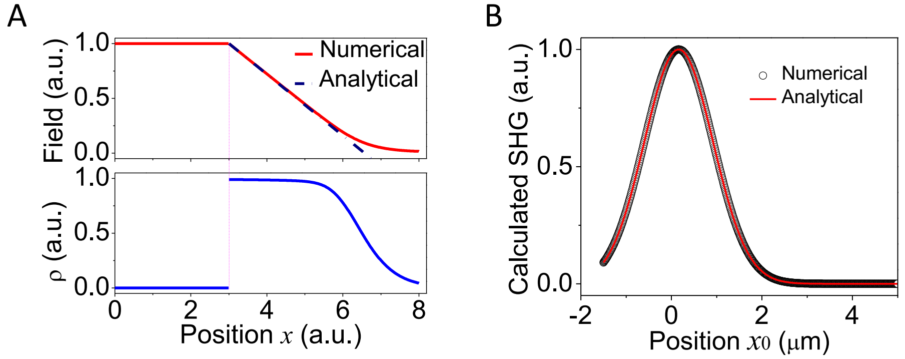

Supplementary Figure 3. **Field profile of high-field domains**. **A**, Typical field profile and space charge distribution when the high-field domain is formed. Here J = -100 Acm^{-2}, donor concentration N_D=5×10^{21} m^{-3}, interface electron density n_c = 0.1N_D, electron mobility μ_n=350 cm^{2}Vs^{-1}, and temperature T=300 K, ε_r=8.9 for CdS [7]**.** The comparisons are also made between the analytical solution under all-depletion approximation and the numerical solution obtained by solving the coupled equations (Supplementary Equations. 4 and 5). The analytically calculated field is in excellent agreement with numerically calculated field except in the tail of depletion region (low field) where the electron density in the conduction band should be considered to contribute to the space charge. **B**, SHG calculated from analytical and numerical fields, corresponding to Fig. 3A and showing our model based on the analytical solution fits to the numerical solution very well.

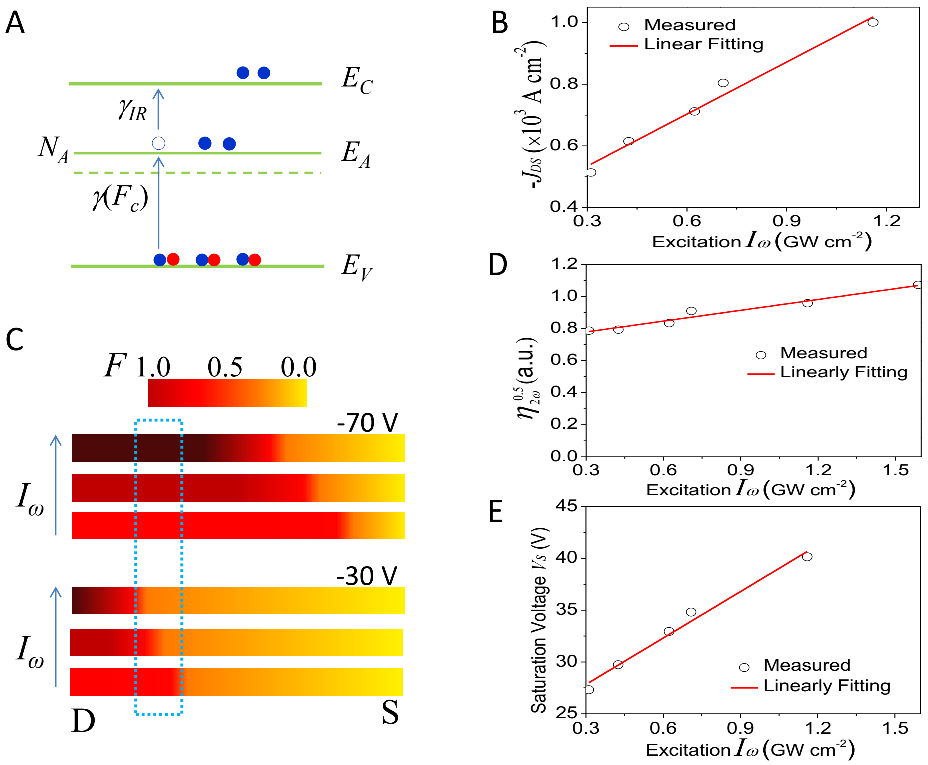

Supplementary Figure 4. Effect of IR quenching on **high-field domains. A**, Schematic of transitions induced by the strong electric field ($\gamma(F_c)$) and IR excitation (γ_{IR}). The blue dots represent electrons while the red dots stand for holes. **B**, Excitation intensity dependence of current at -70 V bias, which shows a linear relationship (Supplementary Equation 22) and is consistent with Fig. 4C. **C**, Distribution of electric field in the CdS nanobelt from the drain (D) contact to source (S) contact under different IR intensity (I_ω), corresponding to Fig. 4C and Fig. 4D. F stands for the electric field in the nanobelt. At -70V, the laser spot is within the high-field domain while it is within or near the domain boundary. The dashed square indicates the laser excitation position. **D**, Linear relationship between $\eta_{2\omega}^{0.5}$ and excitation intensity (I_ω). Given $F_c \propto \eta_{2\omega}^{0.5}$ in Equation 1, the critical field (F_c) for developing a high-field domain is linearly

proportional to the excitation intensity. E, Linear relationship between the saturation voltage (V_s) and excitation intensity (I_ω). V_s is the voltage at which SHG starts to saturate. It was obtained from Fig. 4A.

A

B

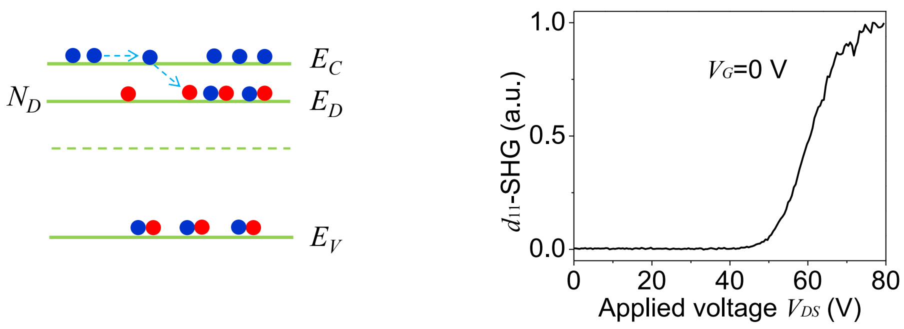

Supplementary Figure 5. Response of **Second Harmonic Generation signal to the applied gate voltage. A**, Schematic of electron redistribution induced by gate voltage via carrier injection. The blue dots represent electrons while the red dots stand for holes. With the application of gate voltage (positive or negative), a large number of electrons can be injected into the semiconductor or extracted, which changes the density of ionized donor traps by the filling or ionizing process. **B**, Voltage-dependent SHG, showing the high-field domain is formed at V_{DS}= 80 V and completely overlaps with the IR excitation.

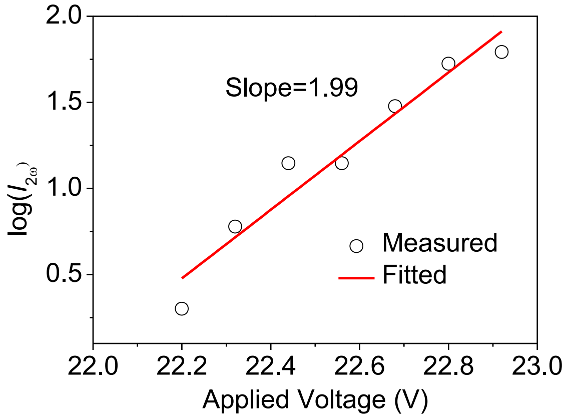

Supplementary Figure 6. The base 10 logarithm of d_{11}-SHG near the threshold voltage where d_{11}-SHG starts to appear. The slope (i.e. $dlogI_{2\omega}/dV$) is about 2, corresponding to the modulation strength of 200%V^{-1}.

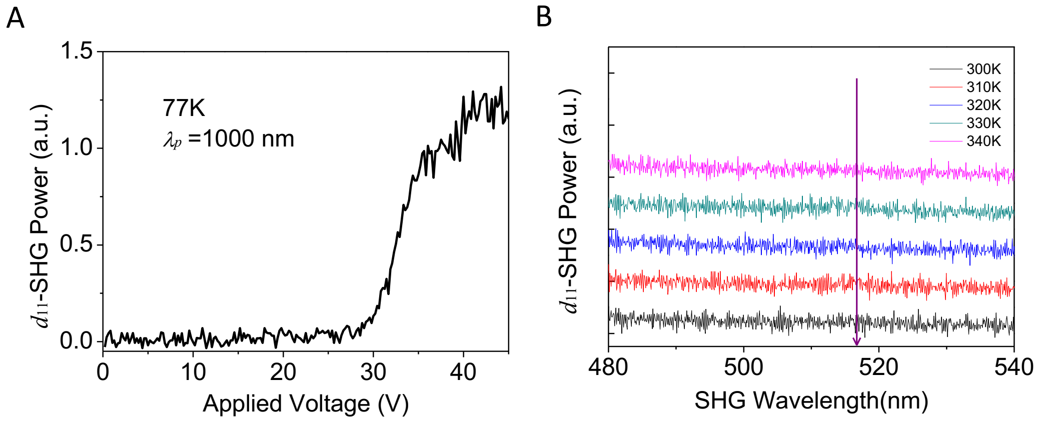

Supplementary Figure 7. No observed heating effect on second-harmonic generation. A. Voltage dependence of d_{11}-SHG at 77K. **B**. Temperature dependence of d_{11}-SHG without applying any bias voltage

Supplementary References

1. Boyd R. W. *Nonlinear optics*, 3rd edn. (Academic Press, 2008).

2. Ren M. L., Agarwal R., Liu W. J., Agarwal R. Crystallographic Characterization of II-VI Semiconducting Nanostructures via Optical Second Harmonic Generation. *Nano Lett.* **15**, 7341-7346 (2015).

3. Böer K. W. *Introduction to space charge effects in semiconductors*. (Springer, 2010).

4. Sze S. M., Ng K. K. *Physics of semiconductor devices*, 3rd edn. (Wiley-Interscience, 2007).

5. Boer K. W. Layer-Like Field Inhomogeneities in Homogeneous Semiconductors in Range of N-Shaped Negative Differential Conductivity. *Phys. Rev.* **139**, A1949-A1959 (1965).

6. Boer K. W., Voss P. Stationary High-Field Domains in Range of Negative Differential Conductivity in Cds Single Crystals. *Phys. Rev.* **171**, 899-903 (1968).

7. Böer K. W. *Electro-optical effects to visualize field and current distributions in semiconductors*. (Springer, 2010).

8. Hussein M., Lleti G., Sagnes G., Bastide G., Rouzeyre M. Deep Level Transient Spectroscopy of Electron Traps and Sensitizing Centers in Undoped Cds Single-Crystals. *J. Appl. Phys.* **52**, 261-268 (1981).

9. Kang L., Cui Y. H., Lan S. F., Rodrigues S. P., Brongersma M. L., Cai W. S. Electrifying photonic metamaterials for tunable nonlinear optics. *Nat. Commun.* **5**, 4680 (2014).

10. Cai W. S., Vasudev A. P., Brongersma M. L. Electrically Controlled Nonlinear Generation of Light with Plasmonics. *Science* **333**, 1720-1723 (2011).

11. Seyler K. L., *et al.* Electrical control of second-harmonic generation in a WSe2 monolayer transistor. *Nat. Nanotechnol.* **10**, 407-411 (2015).

12. Karabulut İ., Baskoutas S. Second and Third Harmonic Generation Susceptibilities of Spherical Quantum Dots: Effects of Impurities, Electric Field and Size. *J. Comput. Theor. Nanos.* **6**, 153-156 (2009).

13. Sugie M., Tada K. Measurements of the Linear Electrooptic Coefficients and Analysis of the Nonlinear Susceptibilities in Cubic GaAs and Hexagonal CdS. *Jpn. J. Appl. Phys.* **15**, 421 (1976).
